# Supplementary figures and images for: Elucidating the role of nicotinamide N-methyltransferase-p53 axis in the progression of chronic kidney disease
Source: PeerJ. 2023 Nov 8;11:e16301. doi: 10.7717/peerj.16301 (PMC10638915; doi:10.7717/peerj.16301)

Figure1

A

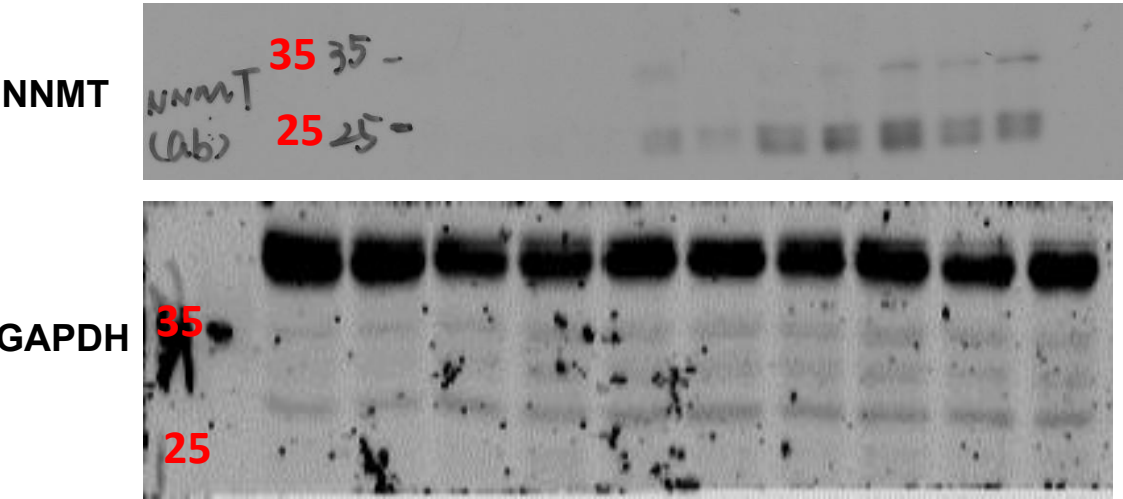

D

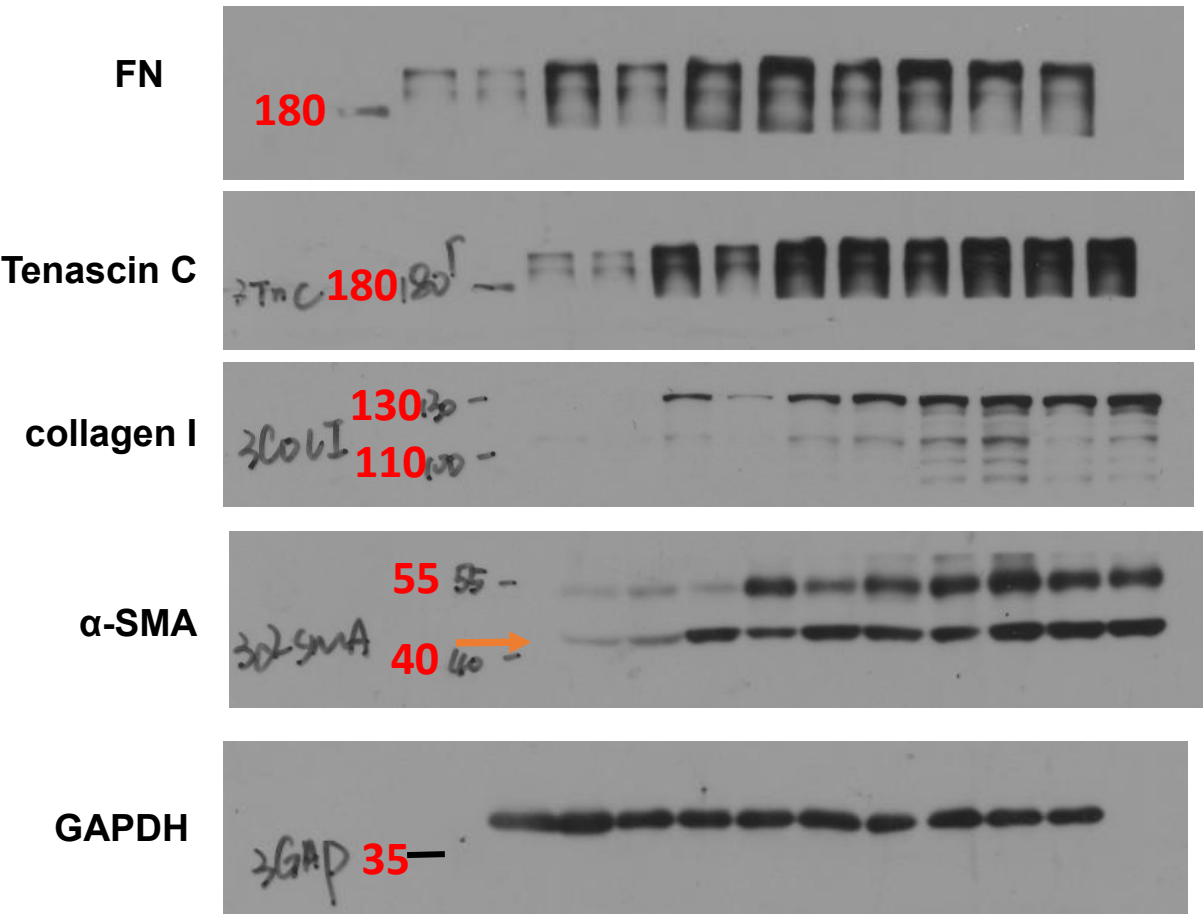

Figure3

A

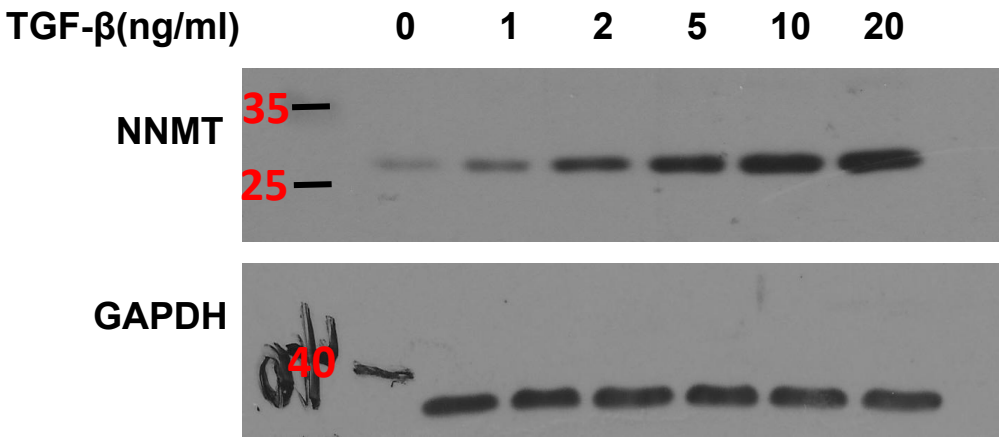

D

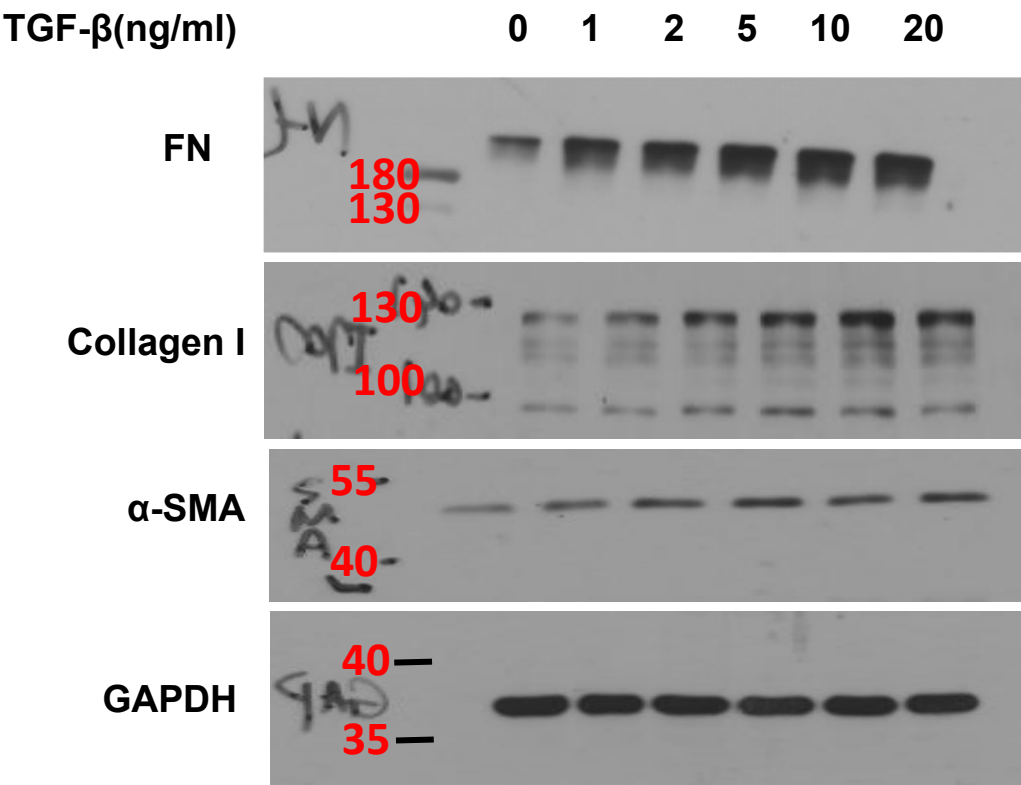

F

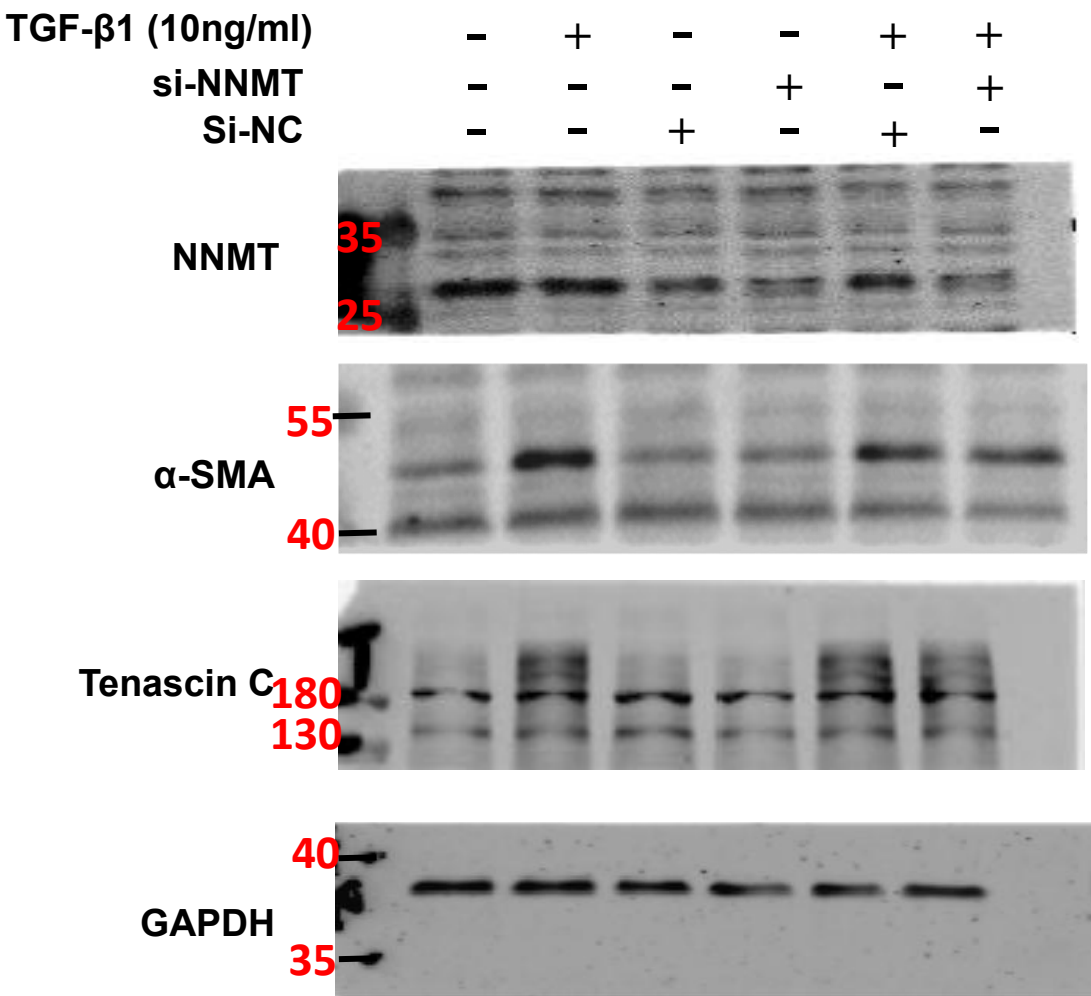

Figure5 A

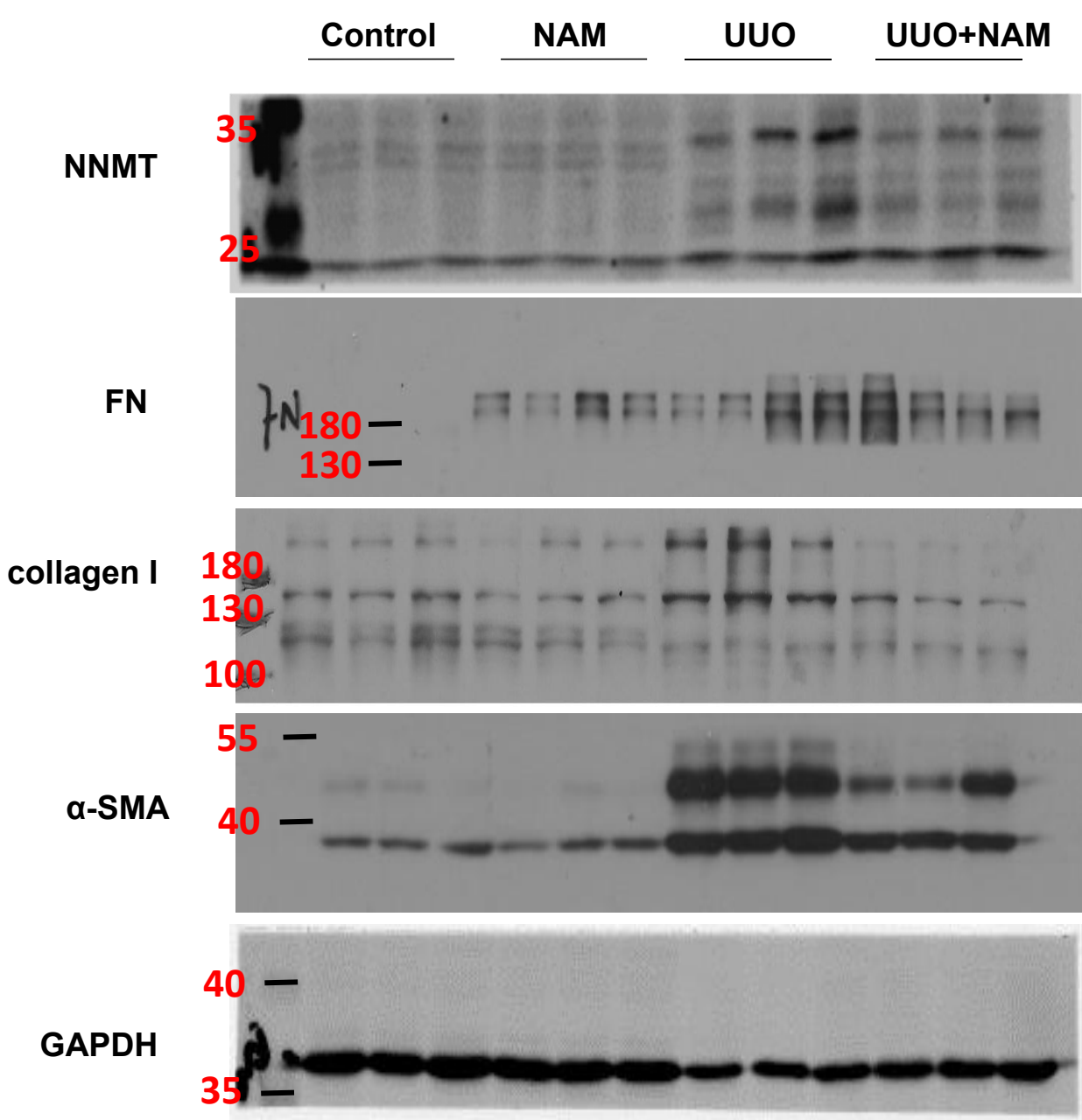

B

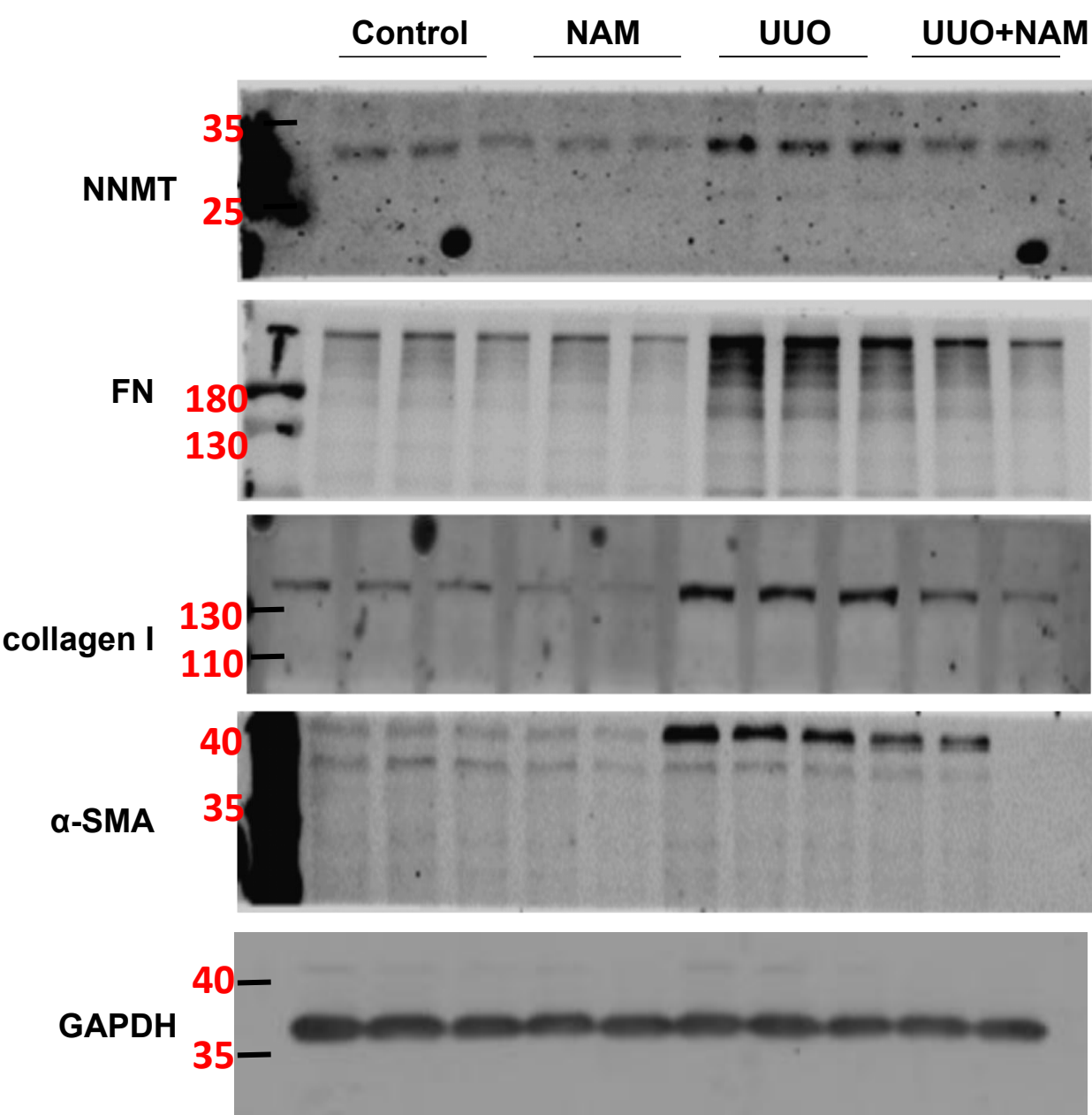

Figure6

B

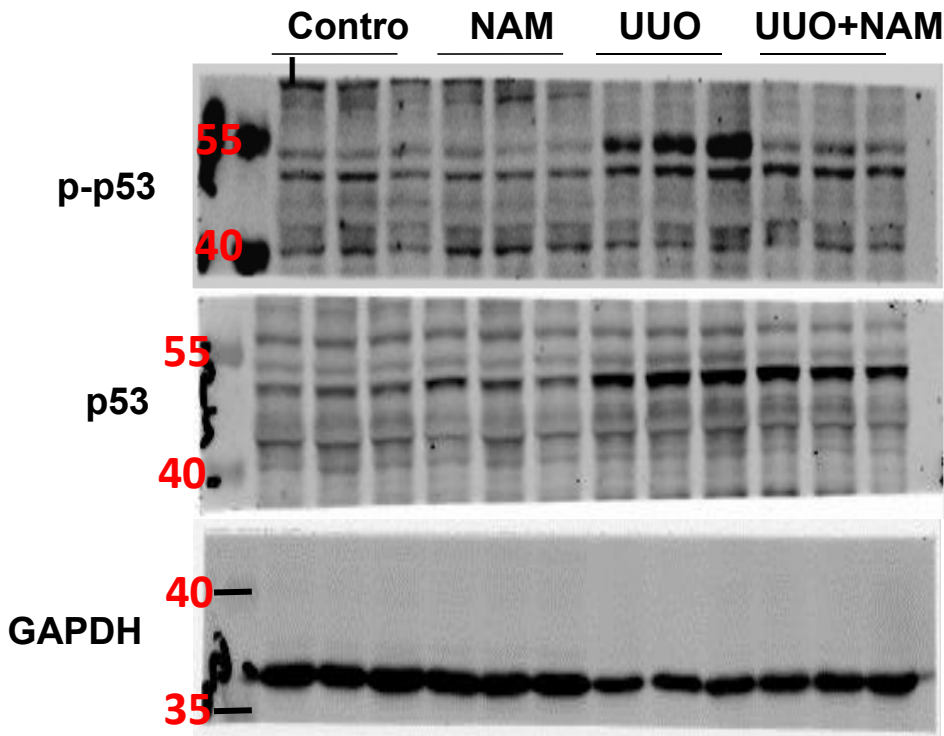

D

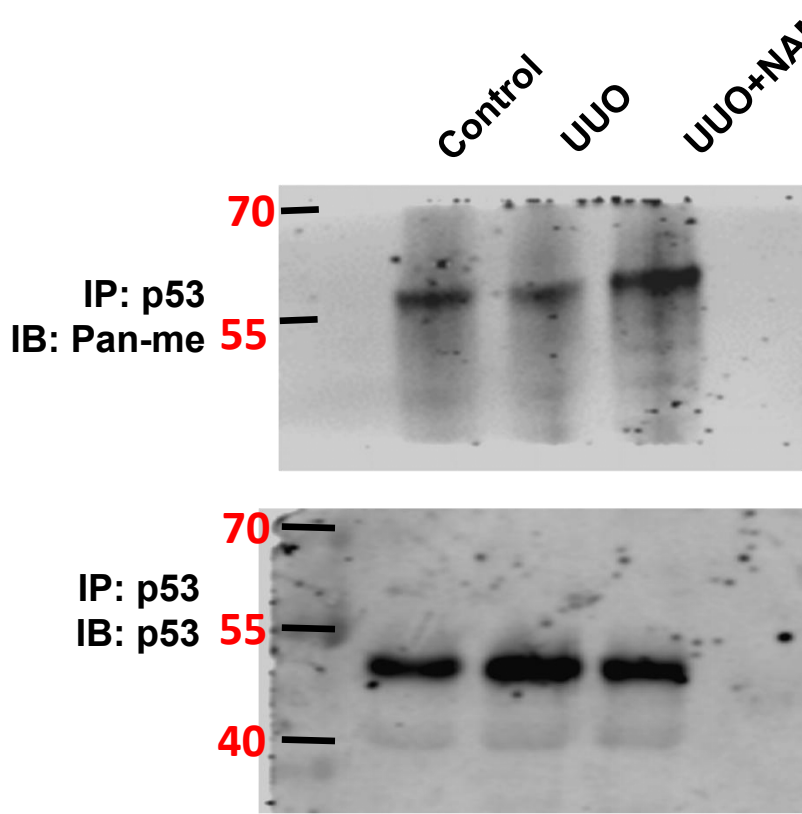

Supplement: Supplemental Information 3 [file peerj-11-16301-s003.pdf]
